# Promoting action control and coping planning to improve hand hygiene

**DOI:** 10.1186/s12889-015-2295-z

**Published:** 2015-09-25

**Authors:** Benjamín Reyes Fernández, Sonia Lippke, Nina Knoll, Emanuel Blanca Moya, Ralf Schwarzer

**Affiliations:** Freie Universität Berlin, Berlin, Germany; Universidad de Costa Rica, San José, Costa Rica; Bremen International Graduate School for Social Sciences (BIGSSS), Bremen, Germany; Institute for Positive Psychology and Education, Australian Catholic University, Sydney, Australia; University of Social Sciences and Humanities, Wroclaw, Poland; Health Psychology, Jacobs Center on Lifelong Learning and Institutional Development (JCLL), Focus Area Diversity, Jacobs University Bremen gGmbH, Campus Ring 1, 28759 Bremen, Germany

## Abstract

**Background:**

We examined a brief educational intervention addressing hand hygiene self-regulatory mechanisms, and evaluated which psychological mechanisms may lead to hand hygiene behaviours.

**Methods:**

Two hundred forty two students (mean age = 21 years, *SD* = 3.9) received either an experimental (*n* = 149) or a control condition on action control and planning (*n* = 93). Hand hygiene, coping planning, and action control were measured at baseline and six weeks later. By applying repeated measures ANOVA, we compared the experimental condition addressing planning to perform hand hygiene with a control condition. Additionally, working mechanisms were evaluated by means of mediation analysis.

**Results:**

The intervention had an effect on action control, as reflected by a time by treatment interaction. The direct effect of the intervention on behaviour was, however, non-significant. Changes in action control led to changes in coping planning. These social-cognitive changes mediated the effect of intervention on behaviour, after controlling for gender, baseline behaviour, and classroom membership.

**Discussion:**

In spite of the associations between the intervention and self-regulatory strategies, no direct effect was found of the intervention on behaviour. Further research on how to increase hand sanitizing, involving enviromental characteristics, is required.

**Conclusion:**

The intervention led only indirectly to an improvement of hand hygiene via changes in self-regulatory factors. Results indicate the importance of promoting action control and coping planning to initiate changes in hand hygienic behaviours.

## Background

*Hand hygiene* contributes to reduced transmission of influenza and acute respiratory tract infection [1] as well as diarrhoea and other infectious diseases [2]. Adequate hand hygiene is regarded as a key measure to prevent health-care associated infections [3]. In spite of that, lack of hand hygiene behaviours seems to be persistent among medical students [4]. Moreover, psychological mechanisms that lead to hand hygiene are not yet well understood [5].

Previous studies have paid little attention to the psychological process underlying hand hygiene behaviours, although recently motivational and volitional processes have been addressed [6]. Also, past research has been conducted among health care workers in hospital settings [7], and other populations, such as *university students*. Replicating effects from behaviour with psychological variables and in university studies deserve attention. Some evidence suggests that hand hygiene is less frequent among younger people [8]. Moreover, studies report that hand hygiene among university students is performed less frequently than desired in key situations, such as before eating or after defecation [9, 10].

The relevance of hand hygiene for students of health-related disciplines is, then, twofold: (1) university campuses and student residences are places where infection transmission might occur more easily, and (2) the acquisition of hand hygiene habits by students might be crucial for their later behaviour in professional settings, where it has consequences not only for their own health but also for clients’ health.

It is important to take into account that hand hygiene can be done by means of alcohol-based antiseptics (hand sanitizer) or by means of soap and water [11]. Alcohol-based hand rubbing removes microorganisms effectively, requiring less time and irritating hands less often than hand washing does with other antiseptic agents and water [12]. Although the use of hand sanitizers is not always recommended over the use of soap and water, it is the measure to take when availability of soap and water is not guaranteed, such as public places or when travelling [13]. Moreover, soap dispensers in public restrooms are frequently contaminated with bacteria at levels much higher than recommended [14], and contaminated bulk-soap-refillable dispensers can lead to bacteria transmission [15]. Soap dispensers in Costa Rican universities are not always in good conditions, but fortunately hand sanitizers are easily accessible in supermarkets and drugstores. Furthermore, hand hygiene by use of hand sanitizer has been found to reduce illness rate in university settings [16].

Given that hand hygiene is a phenomenon of behavioural nature, *psychological variables* should be taken into account when designing interventions: In previous studies, such interventions have been found to be effective (e.g., [6, 17]). To understand health behaviours from a psychological perspective, a self-regulation framework offers an adequate approach. *Self-regulation* refers to any efforts undertaken to alter one’s behaviour [18, 19]. It involves *self-monitoring*, *awareness of standards,* and *effort*, which, working together, have also been conceptualized as *action control* [20]. Action control is considered to be a proximal predictor of behaviour. However, it implies the recall of previously formulated plans.

*Planning* is another factor of self-regulation, reflecting a prospective psychological strategy. Planning is a mental simulation of linking concrete responses to future situations. Using this strategy, the ineffective, spontaneous reactions formed in-situ are replaced by planned responses, which include details of action implementation on how, when, how often, and where to perform the intended behaviour, known as *action plans.* In addition, detailed strategies for coping with anticipated obstacles are known as *coping plans* [21] and are important for behaviour change. When, as part of action control, awareness of standards are activated, then a recall takes place on how and under which circumstances coping strategies should be applied.

Broadly described, psychological variables involved in the health action process approach (HAPA) [22] can be classified as *motivational*, when they lead to the elaboration of behavioural intentions, or *volitional*, when instructions and strategies on how to translate the intention into action take place. Within this theoretical framework, planning and action control are considered volitional variables, which may operate in a sequential manner, either planning preceding action control [23] or action control preceding planning.

For the specific case of hand hygiene, motivational variables have been previously examined in the Costa Rican context [24]. However, the contribution of key volitional variables, and the relationships among them, needs to be further studied. Some studies have examined the role of planning in hand hygiene, although with a very restricted sample size [25], but to our knowledge the role of action control has not yet been explored.

### Aims and hypotheses

A brief educational intervention was designed to examine mechanisms that might play a role in changing hand hygiene, particularly the use of hand sanitizers. It was assumed that the health-enhancing behaviour might be somewhat improved as a result of the brief intervention and that self-regulatory variables, coping planning, and action control, account for individual differences in behaviour. Therefore, the following hypotheses were tested.

1. The intervention will increase the frequency of hand hygiene behaviours.
2. The intervention will produce changes in self-regulatory variables, namely coping planning and action control.
3. Changes in coping planning and action control, specified as mediators, will account for some amount of individual differences in behaviour.

## Methods

### Participants and procedures

University students in Costa Rica (longitudinal analytic sample, *N* = 242), around half of them from health-related disciplines (56 %), took part in an educational experiment. Mean age was 21 years (*SD* = 3.9 years). Most participants were women (61 %), single (97 %), and the majority perceived their health as being good or excellent (78 %).

A sample of 440 students participated at baseline, and 307 of them took part at Time 2 (307 completers, 133 non-completers). Non-completers cited academic duties (field work, meetings) as reasons for drop out. The highest rate of missing values corresponds to Time 2 (T2) behaviour (10.4 %). Due to mismatch, the remaining analytic sample was of *n* = 242 participants.

To avoid spill-over between conditions, classroom groups were randomized to determine whether students received the experimental condition or the control condition. Class lists, provided by the university, were used by researchers to randomise classroom groups (as control variables). Participants remained blind to their allocation during the study. The experiment and data collection were performed between March and November 2014. Participants were recruited over this period of time, and questionnaires were filled out in their classrooms. The questionnaires were completed at baseline and six weeks later.

The study procedures were approved by the ethics committee of the Universidad de Costa Rica. Informed consent was provided by all participants before receiving the baseline questionnaires.

### Experimental and control conditions

Information on how to clean their hands (rubbing palms, back of hands, under fingernails, between fingers) as well as when and in which situations it is needed (before meals and before going to bed, after using the toilet, coughing or sneezing, touching animals, going to public places, after and before travelling, as well as whenever the hands get dirty) was included in an experimental pamphlet.

A planning task was presented, in which participants had to elaborate, based on their everyday life activities, three *action plans* on how often, when, where, and how to clean their hands (e. g., “after meeting my classmates in the library on Wednesday, by applying my hand sanitizer…). They also had to specify *coping plans*, in concrete, what to do to implement their plans in case difficulties appear (e.g., "in case I forget my hand sanitizer, I can buy one in the shop in front of the library after meeting my classmates").

Participants in the experimental condition received, read and filled out the health education pamphlet just after completing the baseline questionnaire. Research assistants were available to supervise the planning task, and to answer questions concerning the intervention and the questionnaire completion.

In the control condition, participants only completed the baseline questionnaire, without any further information pamphlet or task.

### Measures

The study variables were hand hygiene behaviour (use of hand sanitizer), coping planning, and action control, measured at baseline (Time 1; T1) and six weeks later (Time 2; T2). Hand hygiene was measured by the item: “During the past week, I disinfected my hands with hand sanitizer”. Responses followed a 5-point Likert scale, including “0–2”, “3–4”, “5–6”, “7–9”, and “10 or more”, indicating the daily frequency of using disinfectant within one week.

Social-cognitive variables had a 4-point Likert scale response format. Coping planning was measured with three items, such as “To keep my habit in difficult situations, I made a concrete plan for disinfecting my hands, considering what to do when I am in a hurry”. Cronbach’s alpha was .82 at T1 and .88 at T2. Action control was measured with three items, such as “During the week, I watched consistently when, how often, and how to disinfect my hands”. Cronbach’s alpha was .78 at T1 and .81 at T2.

Change scores for the social-cognitive variables were computed by subtracting T1 scores from T2 scores.

### Analysis

Statistical analyses were performed with SPSS 22. Drop-out analyses were performed by means of t-tests for continuous variables and χ^2^ for categorical variables, in order to compare the retained and lost individuals at T2. Randomization checks were conducted between participants of the control and the experimental conditions. MANOVA was used to test the baseline differences for continuous variables, and χ^2^ tests were used for categorical variables. Intervention effects were examined by means of repeated measures ANOVA. Psychological mechanisms were assessed in terms of serial mediation with the SPSS PROCESS macro by Hayes [26]. In serial mediations multiple mediators are assumed to operate sequentially in a causal chain, from an independent variable, through more than one mediator, and concluding in a final consequent variable. In the present case changes in action control and changes in coping planning, in this order, were specified as sequential mediators between the intervention and T2 hand hygiene behaviour. To control for classroom effects, classroom was specified as a control variable using the fixed effects approach (see e.g., Cohen et al., 2003, pp. 539–544). In this approach the control variables are dummy coded to partial out their effects in the model. Gender and baseline behaviour were included as covariates.

## Results

### Drop-out analysis and randomization checks

From the original sample (*n* = 440) 307 were completers, and 133 were non-completers. Non-completers cited academic duties (field work, meetings) as reasons for drop out. Those who completed the study had slightly higher coping planning levels at baseline, *t*(424) = −2.19, *p* = .03, Cohen’s *d* = −.24, (*M*_completers_ = 2.27, *SD*_completers_ = 0.92; *M*_non-completers_ = 2.05, *SD*_non-completers_ = 0.88). No baseline differences were found for gender, age, action control, and baseline hand hygiene behaviour between those who completed the study and those who did not.

Concerning the randomization, no differences at baseline were found for coping planning, age, and gender. However, for action control, the group which received the control condition presented slightly higher baseline levels than the group receiving the experimental condition (*M*_control_ = 2.82, *SD*_control_ = 0.79; *M*_experiment_ = 2.54, *SD*_experiment_ = 0.90; *F*(1,240) = 6.205, *p* = .01, Cohen’s *d* = 0.33), and for hand hygiene behaviour, the group in the control condition reported lower levels than the group in the intervention condition (*M*_control_ = 1.46, *SD*_control_ = 0.88; *M*_experiment_ = 1.84, *SD*_experiment_ = 1.32; *F*(1,240) = 5.918, *p* = .01, Cohen’s *d* = 0.32).

### Experimental effects

Table 1 contains the means and standard deviations for each variable as well as group comparison statistics at T1 and T2 for both conditions. Baseline differences between experimental and control groups existed for behaviour (in favour of the experimental group) and action control (in favour of the control group). At follow up, differences between experimental and control groups remained for behaviour (in favour or the experimental group). The difference in action control was still visible but not statistically significant. Analysing time and interaction effects, the following patterns resulted. No interaction between treatment and time was found. However, there was an effect of time on behaviour, *F*(1,243) = 7.74, *p* = .006, η^2^ = .03. In other words, behaviour was increased in both groups significantly. For action control, there was an interaction between treatment and time, *F*(1,243) = 11.01, *p* = .001, η^2^ = .04. For coping planning, no substantial effect was found neither for time nor for the interaction of treatment and time, although the interaction term was marginally significant, *F*(1,239) = 2.96, *p* (2-tailed) = .045, η^2^ = .01.

**Table 1:** Means and Standard Deviations (SDs) of hand hygiene, action control and coping planning at pre-test and at post-test, and comparison between experiment conditions

**Measurement time**	**Variable**	**Condition**	**M**	**SD**	**T**	***p***	***D***
**Pre-test**	**Hand hygiene behaviour**	Control	**1.46**	**0.88**	**−2.662**	**.008**	**−.34**
		Experimental	**1.84**	**1.32**			
	**Action control**	Control	**2.82**	**0.79**	**2.491**	**.013**	**.33**
		Experimental	**2.54**	**0.90**			
	**Coping planning**	Control	2.34	0.92	.562	.575	.07
		Experimental	2.27	0.95			
**Post-test**	**Hand hygiene behaviour**	Control	**1.74**	**1.29**	**−1.700**	**.090**	**-.23**
		Experimental	**2.05**	**1.39**			
	**Action control**	Control	2.72	0.86	−.843	.400	−.20
		Experimental	2.89	0.84			
	**Coping planning**	Control	2.33	1.00	−1.064	.288	−.13
		Experimental	2.46	0.94			

Note. Longitudinal sample *N* = 242. Listwise deletion. Bold numbers are used for variables for which the comparison statistics are *p* (2-tailed) < .05

**Fig. 1:**
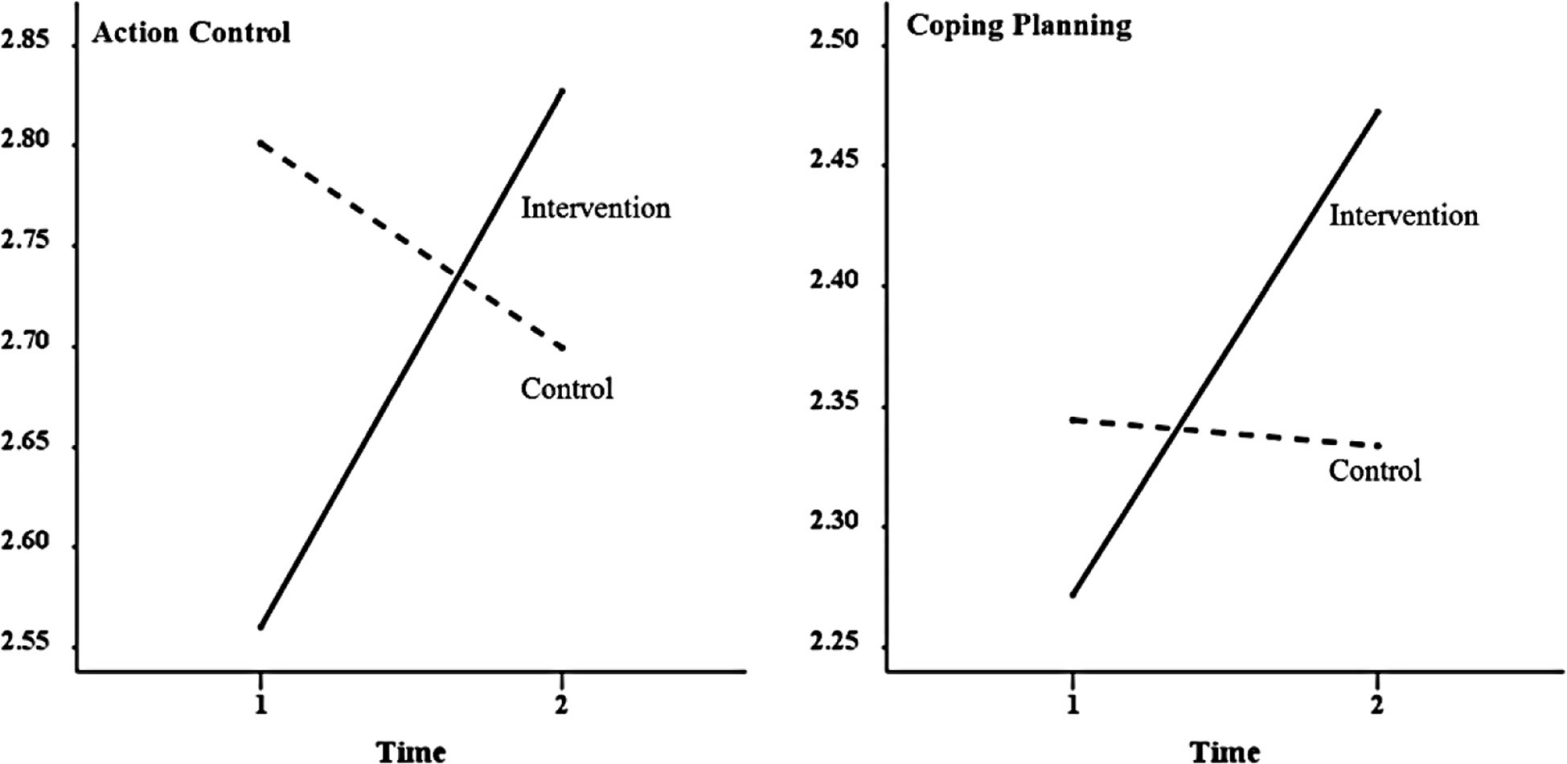
Levels of action control and coping planning in the two experimental conditions at two points in time

The means for action control and coping planning for the two groups and at T1 and T2 are depicted in Fig. 1. The response options for action control and for coping planning define that 3 is the threshold from which there is an agreement with the statements, namely, that action control has taken place and that plans were elaborated. As can be seen in Fig. 1, the mean responses for both social-cognitive variables do not exceed 3. The experimental group increased their means over time whereas the control group decreased or maintained its mean level. Thus, the experiment resulted in a clear increase in social-cognitive variables in comparison to the control condition.

### Mediation analysis

**Fig. 2:**
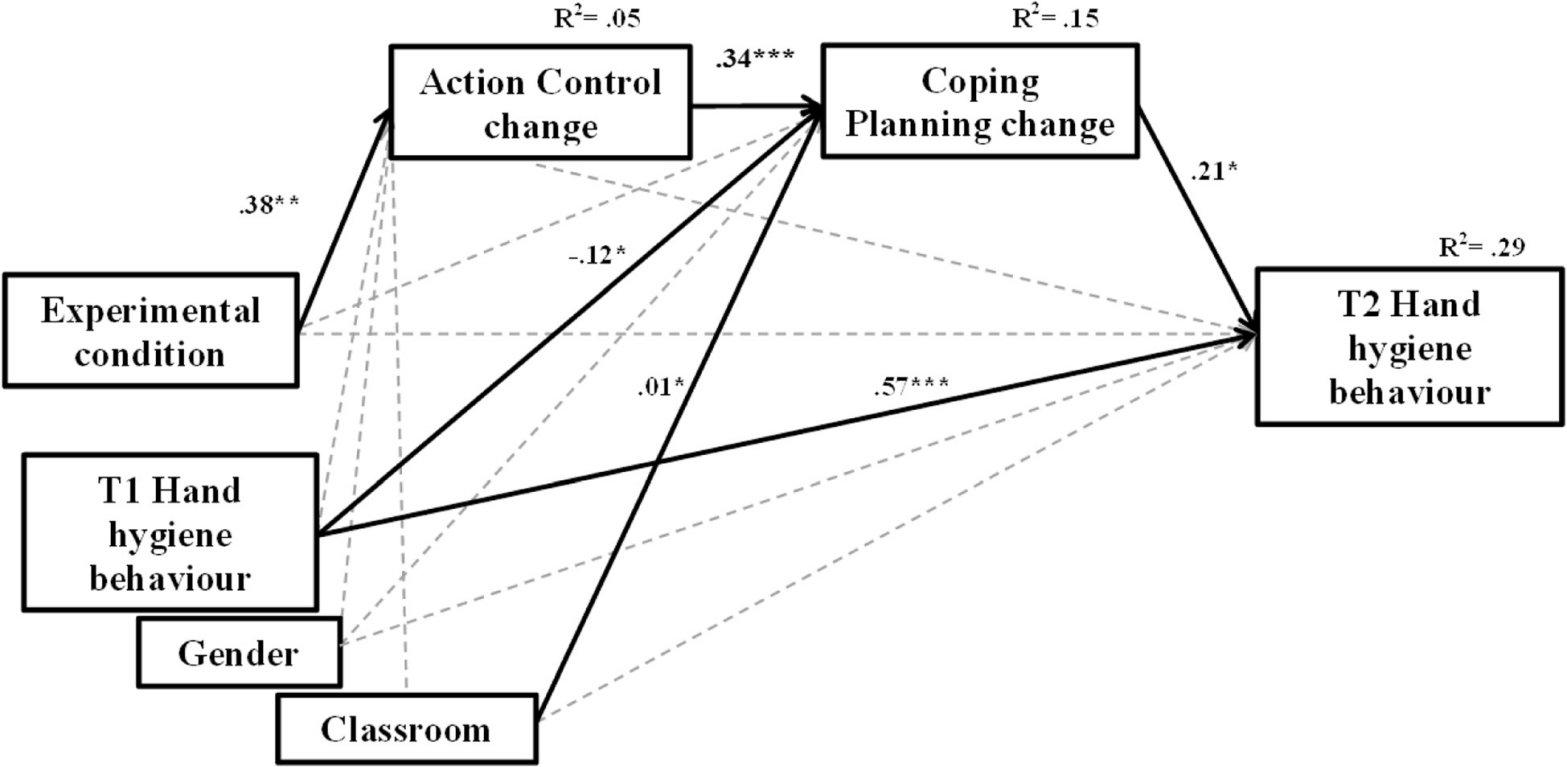
Indirect serial effects of the experimental condition on hand hygiene behaviour via changes in action control and changes in coping planning, controlling for the effects of baseline behaviour, gender and control variable classroom on mediators and on the outcome. Unstandardized solution, bootstrapped with 5,000 resamples. *N* = 242. ***p < .001, **p < .01, *p < .05

The serial mediation analysis addressed the question on how social-cognitive variables (operationalizing the behaviour change strategies) contribute to elucidate the working mechanisms underlying the experimental effects. Results are depicted in Fig. 2.

The experimental condition had an effect on the action control change score, *b* = .38, CI 95 % [.15, .61]. Action control change had an effect on coping planning change, *b* = .34, CI 95 % [.21, .47]. Subsequently, coping planning change had an effect on T2 hand hygiene behaviour, *b* = .21, CI 95 % [04, .38].

Gender as a covariate and classroom as a control variable were not associated with T2 behaviour. Baseline behaviour had an effect on coping planning change, *b* = −.12, CI 95 % [−.22, −.03]. Classroom had a significant but lower correlation with coping planning change, *b* = .01, CI 95 % [.00, .02]. The total indirect effect was *b* = .08, CI 95 % [.00, .20], and the indirect effect chain intervention → action control change → coping planning change → T2 behaviour was *b* = .03, CI 95 % [.00, .08], suggesting that the indirect effect followed a sequence including all the mediators. Thus, the variance found at the level of behaviour is basically attributable to the chain involving cognitive variables rather than to gender or classroom characteristics.

## Discussion

Proper hand hygiene is imperative for preventing the spread of different diseases and, when there is no adequate soap available, the use of hand sanitizers has been found as an adequate alternative [27]. Previous studies have shown that most students do not perform the recommended behaviours at a sufficient level [4]. Therefore, this study investigated whether a brief educational intervention could increase social-cognitive predictors of hand hygiene behaviour as well as hand hygiene itself. The brief intervention produced changes in social-cognitive variables, confirming the corresponding hypothesis (Hypothesis 2). It was sufficiently powerful to eliminate the difference found at baseline between conditions in action control. However, it was not sufficient to produce changes in behaviour over time, disproving the behavioural hypothesis (Hypothesis 1).

Social-cognitive variables stayed at a low and, practically speaking, at a “non-implementation” level. In the response format of the items used, a score of three or more means that the participant has elaborated plans or that he or she has performed action control strategies. Even though there was an increment in social-cognitive variables in the intervention condition, it did not surpass the minimum level of 3. Thus, on average changes in social-cognitive variables were not enough to produce changes in hand hygiene behaviour over time.

Volitional variables, although frequently conceptualized in a temporal sequence, may work altogether as part of a self-regulatory mechanism, and, thus, some effects of putative posterior variables on putative precedent variables could be expected. This was certainly found in the action control-planning relationship, where the former received effects from the last one, as suggested in the mediation analysis. By activating the self-regulatory strategies of action control, awareness of previously elaborated plans increases and then the cue-response link may become stronger. Therefore an intervention on planning may increase planning via action control and, subsequently, behaviour, although these changes may not be sufficient to produce an interaction between time and treatment in hand hygiene behaviour. However, there was certainly a mediation of social-cognitive variables between the intervention and behaviour, confirming hypothesis 3. Practically speaking, those study participants in the experimental group increasing action control and coping planning due to the intervention were also more likely to perform disinfection behaviour. This matched previous findings, documenting that educational interventions can change psychological outcomes and by these means also behaviours (*e.g*., [6, 17]).

There are some limitations in this study. Assessments were self-reported, and hand hygiene was measured retrospectively. Retrospective methods are vulnerable to unintentional misreporting (e.g., due to recall errors).

This could be overcome by using concurrent direct observation, where observers are trained to assess the quality and quantity of hand hygiene behaviours [28]. However, such a measurement strategy is resource demanding and requires the existence of a closed setting, such as a hospital, where all possible occurrences of behaviour take place in a limited observable physical place. For university students, who could get in or out of the campus, this is hardly feasible.

Furthermore, the current study applied only a very brief intervention including only action planning and coping planning. In future studies, motivational constructs could be addressed (such as convincing students first, that the use of hand sanitizer is effective in preventing illness) and as well as other volitional variables (such as action control or self-efficacy). Additionally, although cluster randomization might have some advantages over randomization at the individual level [29], the reduced number of cluster units is a limitation, and may have contributed to the baseline differences found for behaviour and action control. A larger number of cluster units, either classrooms, universities, or communities, should be included for further research.

## Conclusion

In conclusion, the present study explored the behaviour change strategies (planning and action control) that are thought to translate intervention content into behavioural outcomes [30, 31]. The current intervention documented effects on these putative mediators but failed to result in visible changes in hand hygiene behaviours. This can be due to the parsimony of the treatment or to environmental factors, such as availability of products for hand hygiene [32], which were not assessed in the current study. Recommendations from this study are: Further theory-guided educational interventions should be used testing psychological mechanisms, which may enable more behaviour change. Thus, to increase hand hygiene behaviour, concrete planning of when, where, and how to disinfect one’s hands, and how to deal with barriers should be facilitated.

## Abbreviations

ANOVA: Analysis Of Variance
B: Unstandardized coefficient
CI: Confidence intervals
D: Cohen’s d (Effect size)
e.g.: exempli gratia/ for example
F: F-statistic from F-test/ ANOVA
χ^2^: Chi squared Test
η^2^: Eta squared (Effect size)
M: Mean
MANOVA: Multiple Analyses Of Variance
N: Sample size
P: p-value
R^2^: R squared (Explained Variance)
SD: Standard deviation
SPSS: Statistical package for the social sciences software
T: t-Statistic from t-Test
T1: Measurement point time 1
T2: Measurement point time 2
